# Ultrastructural Analysis of Cell Envelope and Accumulation of Lipid Inclusions in Clinical *Mycobacterium tuberculosis* Isolates from Sputum, Oxidative Stress, and Iron Deficiency

**DOI:** 10.3389/fmicb.2017.02681

**Published:** 2018-01-11

**Authors:** Srinivasan Vijay, Hoang T. Hai, Do D. A. Thu, Errin Johnson, Anna Pielach, Nguyen H. Phu, Guy E. Thwaites, Nguyen T. T. Thuong

**Affiliations:** ^1^Oxford University Clinical Research Unit, Ho Chi Minh City, Vietnam; ^2^Centre for Tropical Medicine and Global Health, Nuffield Department of Medicine, University of Oxford, Oxford, United Kingdom; ^3^Sir William Dunn School of Pathology, University of Oxford, Oxford, United Kingdom; ^4^Hospital for Tropical Diseases, Ho Chi Minh City, Vietnam

**Keywords:** *Mycobacterium tuberculosis*, ultrastructure, intracytoplasmic lipid inclusions, cell envelope, oxidative stress, iron deficiency and mesosome

## Abstract

**Introduction:** Mycobacteria have several unique cellular characteristics, such as multiple cell envelope layers, elongation at cell poles, asymmetric cell division, and accumulation of intracytoplasmic lipid inclusions, which contributes to their survival under stress conditions. However, the understanding of these characteristics in clinical *Mycobacterium tuberculosis* (*M. tuberculosis*) isolates and under host stress is limited. We previously reported the influence of host stress on the cell length distribution in a large set of clinical *M. tuberculosis* isolates (*n* = 158). Here, we investigate the influence of host stress on the cellular ultrastructure of few clinical *M. tuberculosis* isolates (*n* = 8) from that study. The purpose of this study is to further understand the influence of host stress on the cellular adaptations of clinical *M. tuberculosis* isolates.

**Methods:** We selected few *M. tuberculosis* isolates (*n* = 8) for analyzing the cellular ultrastructure *ex vivo* in sputum and under *in vitro* stress conditions by transmission electron microscopy. The cellular adaptations of *M. tuberculosis* in sputum were correlated with the ultrastructure of antibiotic sensitive and resistant isolates in liquid culture, under oxidative stress, iron deficiency, and exposure to isoniazid.

**Results:** In sputum, *M. tuberculosis* accumulated intracytoplasmic lipid inclusions. In liquid culture, clinical *M. tuberculosis* revealed isolate to isolate variation in the extent of intracytoplasmic lipid inclusions, which were absent in the laboratory strain H37Rv. Oxidative stress, iron deficiency, and exposure to isoniazid increased the accumulation of lipid inclusions and decreased the thickness of the cell envelope electron transparent layer in *M. tuberculosis* cells. Furthermore, intracytoplasmic compartments were observed in iron deficient cells.

**Conclusion:** Our ultrastructural analysis has revealed significant influence of host stress on the cellular adaptations in clinical *M. tuberculosis* isolates. These adaptations may contribute to the survival of *M. tuberculosis* under host and antibiotic stress conditions. Variation in the cellular adaptations among clinical *M. tuberculosis* isolates may correlate with their ability to persist in tuberculosis patients during antibiotic treatment. These observations indicate the need for further analyzing these cellular adaptations in a large set of clinical *M. tuberculosis* isolates. This will help to determine the significance of these cellular adaptations in the tuberculosis treatment.

## Introduction

*Mycobacterium tuberculosis* (*M. tuberculosis*), causes tuberculosis (TB) and is a major public health problem (World Health Organization [WHO], 2015). The ability of *M. tuberculosis* cells to survive under host and antibiotic stress partly explains why *M. tuberculosis* is a successful human pathogen. Hence, cellular adaptations conferring stress tolerance in *M. tuberculosis* and in related species are an active area of research (Kieser and Rubin, 2014).

Investigations into cell biology of mycobacteria have revealed several unique characteristics in growth and division, which contributes to their survival under stress conditions (Thanky et al., 2007; Hett and Rubin, 2008; Kieser and Rubin, 2014). One such cellular structure is the complex cell envelope of mycobacteria (Brennan and Nikaido, 1995). Electron microscopy has revealed the ultrastructure of cell envelope layers in mycobacteria (Takade et al., 1983; Hoffmann et al., 2008; Zuber et al., 2008; Vijay et al., 2012). The cell envelope is essential for *M. tuberculosis* survival as it acts as a permeability barrier for the entry of antibiotics and also modulates host immune response (Jarlier and Nikaido, 1994; Briken et al., 2004; Torrelles and Schlesinger, 2010). Therefore, it is also an important drug and vaccine target (Chatterjee, 1997; Abrahams and Besra, 2016; Tima et al., 2017). The composition of cell envelope layers has been determined using cell envelope mutants (Etienne et al., 2002, 2005) and antibiotic treatments which inhibit the envelope synthesis in mycobacteria (Mdluli et al., 1998). These studies have advanced our understanding of the cell envelope role as a permeability barrier and in inhibiting phagocytosis of mycobacteria by macrophages (Mdluli et al., 1998; Etienne et al., 2002, 2005).

Another feature revealed by electron microscopy was the accumulation of intracytoplasmic lipid inclusions in mycobacteria under different host infection model systems (Peyron et al., 2008; Caire-Brandli et al., 2014; Barisch and Soldati, 2017a). In an *in vitro* human granuloma model of infection, *M. tuberculosis* cells accumulated lipid inclusions during infection of lipid loaded macrophages called foam cells (Peyron et al., 2008). Similarly, *M. avium* accumulated host-derived lipids as inclusions in foam cells and exhibited a thin cell envelope (Caire-Brandli et al., 2014). Recently, *M. marinum* was also found to have lipid inclusions derived from host lipids during the infection of *Dictyostelium* (Barisch and Soldati, 2017a). These studies have identified triacylglycerols as the major lipid in mycobacterial lipid inclusions derived from host cells (Peyron et al., 2008; Daniel et al., 2011; Caire-Brandli et al., 2014; Barisch and Soldati, 2017a). *M. tuberculosis* and *M. smegmatis* can also accumulate lipid inclusions containing triacylglycerols under *in vitro* stress conditions independent of host cells (Garton et al., 2002; Anuchin et al., 2009; Deb et al., 2009). Several studies have shown that *M. tuberculosis* uses diverse host carbon sources such as cholesterol, pyruvate, and glucose (Pandey and Sassetti, 2008; Marrero et al., 2013; Baker et al., 2014). Utilization of such diverse carbon sources by *M. tuberculosis* contributes to its pathogenesis and persistence in the host (Pandey and Sassetti, 2008; Marrero et al., 2013; Baker et al., 2014).

Importantly, the accumulation of lipid inclusions in *M. tuberculosis* was associated with persistence, antibiotic tolerance, cavitation, and poor treatment outcome (Deb et al., 2009; Russell et al., 2009; Daniel et al., 2011; Hammond et al., 2015; Kayigire et al., 2015; Sloan et al., 2015). It is possible that this is due to growth arrest of *M. tuberculosis* and loss of antimicrobial functions by foamy macrophages leading to persistent infection (Peyron et al., 2008; Daniel et al., 2011; Caire-Brandli et al., 2014). This phenomenon may lead to clinical complications, such as relapse of infection and the emergence of antibiotic-resistant *M. tuberculosis* (Cohen et al., 2013; Sebastian et al., 2017). Thus, intracytoplasmic lipid inclusions and the cell envelope are important for the survival of *M. tuberculosis.* The understanding of these cellular characteristics and their adaptations to stress in clinical *M. tuberculosis* isolates is limited. This understanding is vital for the development of novel therapeutic targets. In our previous study, we have observed that host stresses influenced cell length distribution in a large set (*n* = 158) of clinical *M. tuberculosis* isolates (Vijay et al., 2017). In this study we investigated the accumulation of lipid inclusions and cell envelope ultrastructure of *M. tuberculosis* in sputum by transmission electron microscopy (TEM). The ultrastructure of *M. tuberculosis* in sputum was compared with the ultrastructure of clinical *M. tuberculosis* isolates and H37Rv in liquid culture, and under conditions of oxidative stress, iron deficiency, and exposure to the antibiotic isoniazid.

## Materials and Methods

### Bacterial Isolates

Six *M. tuberculosis* clinical isolates were selected from a collection of *M. tuberculosis* clinical isolates from pre-treated patients with pulmonary tuberculosis (*n* = 158) in Vietnam, along with the laboratory strain H37Rv. We selected three sensitive and three antibiotic-resistant isolates as determined by drug susceptibility test for the electron microscopy analysis. **Table 1** presents drug sensitivity data.

**Table 1:** *Mycobacterium tuberculosis* clinical strains selected for the study based on antibiotic sensitive and resistant phenotypes.

Strain name	Antibiotic resistance	*M. tuberculosis* lineages
C1	Sensitive	Indo-Oceanic
C2	STR, RIF	ND
C3	Sensitive	Indo-Oceanic
C4	STR	East Asian
C5	Sensitive	East Asian
C6	STR, RIF, INH, EMB	East Asian
H37Rv	Sensitive	Euro American

*STR, streptomycin; RIF, rifampin; INH, isoniazid; EMB, ethambutol, ND, not determined.*

### Ethics Approval Statement

Between January 2015 and October 2016, patients were recruited from two district TB control units in Ho Chi Minh City (HCMC), Vietnam. The clinical *M. tuberculosis* isolates were collected from patients before treatment. The patients were ≥18 years of age, had clinical symptoms of active pulmonary TB, which was confirmed by chest X-ray and positive sputum culture, and none of the patients were HIV positive. Written informed consent was obtained from each patient in accordance with the declaration of Helsinki. Protocols were approved by the human subjects review committees, at the Hospital for Tropical Diseases HCMC, Vietnam (124/BVBNƉ.HƉƉƉ) and the Oxford Tropical Research Ethics Committee, United Kingdom (OxTREC Reference: 16-14).

### Bacterial Culture

*Mycobacterium tuberculosis* isolates were cultured from sputum samples in bio safety level-3 laboratory and were stored as glycerol stocks in 7H9 media. These *M. tuberculosis* isolates were used for the experiments with a limited number of sub-culturing (approximately two to three passages) to avoid phenotypic/genotypic changes in clinical *M. tuberculosis* isolates. For mid-log culture, 50 ml culture tubes with 10 ml of 7H9T medium [7H9 broth supplemented with 10% oleic acid/albumin/dextrose/catalase (OADC) enrichment, and 0.05% Tween 80, BD Difco^TM^] were inoculated with the clinical isolates and laboratory strain H37Rv, incubated at 37°C without shaking. The samples were processed for TEM at O.D_600_ of 0.3–0.6.

### Drug Susceptibility Test

Drug susceptibility was performed using BACTEC^TM^ MGIT^TM^ 960 SIRE Kit (BD), according to manufacturer guidelines. Drug susceptibility was tested for streptomycin (1.0 μg/ml), isoniazid (0.1 μg/ml), rifampicin (1.0 μg/ml), and ethambutol (5.0 μg/ml).

### *M. tuberculosis* Lineage Identification

The lineages of the selected clinical *M. tuberculosis* isolates were determined in the previous study (Vijay et al., 2017).

### Oxidative Stress, Iron Deficiency, and Isoniazid Treatment

For TEM analysis of *M. tuberculosis* cells under different stress conditions, *M. tuberculosis* culture in 7H9T medium at O.D_600_ 0.3–0.5 was treated with H_2_O_2_ (Merk) at different concentrations, ranging from 21 to 210 mM for 48 h at 37°C and selected 21 mM H_2_O_2_-treated samples for electron microscopy (Voskuil et al., 2011). For iron deficiency, *M. tuberculosis* isolates were cultured in the presence of deferoxamine mesylate salt (DFO) (Sigma–Aldrich) at final concentrations of 100, 250, and 500 μM in 7H9T medium until the O.D_600_ reached 0.3–0.5, with the 100 and 500 μM DFO-treated samples processed for electron microscopy (Pal et al., 2015). For isoniazid treatment, *M. tuberculosis* isolates were grown in the presence of isoniazid (Sigma–Aldrich) in 7H9T medium at a concentration of 0.015 μg/ml until the O.D_600_ reached 0.3–0.5. All treated and untreated control isolates, along with about 500 μl of sputum with high density of acid fast bacilli (3+) as observed by microscopy from two pulmonary tuberculosis patients, were then processed for TEM.

### Transmission Electron Microscopy

*Mycobacterium tuberculosis* cells were fixed as described previously (Vijay et al., 2012). *M. tuberculosis* cells were harvested by centrifugation and fixed in 1% (vol/vol) osmium tetroxide (Sigma–Aldrich) and 0.15 M sodium cacodylate buffer (pH 7.2) (Sigma–Aldrich) for 1 h at room temperature. After this samples were washed once with the same buffer, and post fixed for 2 h at room temperature in 0.15 M cacodylate buffer (pH 7.2) containing 2% (wt/vol) tannic acid and 2% (vol/vol) glutaraldehyde (both from Sigma–Aldrich). Samples were then washed once with 0.15 M cacodylate buffer and then refixed in 1% (vol/vol) osmium tetroxide overnight at 4°C and stored at 4°C for 2–4 weeks before further processing. Next the samples were washed with water and cells were re-suspended in 4% low melting point agarose, spun down, and stored at 4°C for few minutes. These samples were cut into small fragments of less than 1 mm^3^ and stained with 0.5% uranyl acetate overnight and washed with water. Subsequent steps were performed using a Leica EM TP automated processing unit (Leica Microsystems). Samples were dehydrated in a graded series of ice cold ethanol (Merck) and then infiltrated with epoxy resin (Taab Low Viscosity Resin, Taab Laboratories) as follows: 25% resin in ethanol for 2 h, 50% resin for 3 h, 75% resin for 2 h, then 100% resin over 48 h with several changes of resin. Samples were polymerized in beem capsules at 60°C for 48 h. Ultrathin sections (90 nm) were obtained using a Leica UC7 Ultramicrotome and a Diatome Diamond Knife (Leica microsystems and Diatome). Sections were transferred to formvar coated 100 mesh Cu grids and post-stained with Reynolds’ lead citrate (Reynolds, 1963). Sections were imaged on an FEI Tecnai 12 Transmission Electron Microscope operated at 120 kV using a Gatan OneView digital camera. In each condition approximately 100 *M. tuberculosis* cells per sample were observed, except sample S2 (*n* = 10 cells). Cell envelope layer measurements were carried out using ImageJ (Schneider et al., 2012).

## Results

### *M. tuberculosis* in Sputum Displayed Triple Layered Cell Envelope and Accumulation of Intracytoplasmic Lipid Inclusions

**FIGURE 1:**
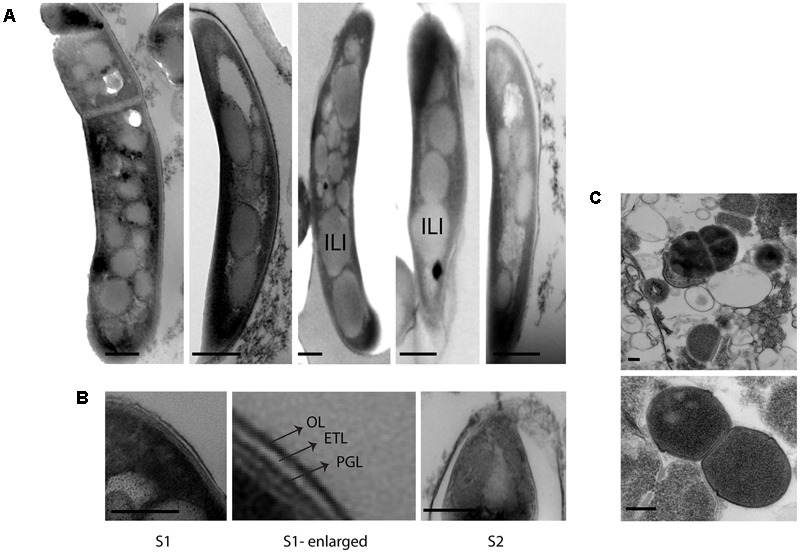
Cellular ultrastructure of *M. tuberculosis* cells from patient sputum samples. **(A)** TEM images of *M. tuberculosis* cells with intracytoplasmic lipid inclusions in sputum sample S1. **(B)** TEM images of *M. tuberculosis* cells with detailed ultrastructure of cell envelope layers OL, ETL, and PGL from two different sputum samples S1 and S2. **(C)** TEM images of other bacterial cells in sputum sample S1. OL, outer layer; ETL, electron transparent layer; PGL, peptidoglycan layer; ILI, intracytoplasmic lipid inclusions; TEM, transmission electron microscopy. Scale bar = 200 nm.

Initially, we investigated *M. tuberculosis* cell envelope ultrastructure and lipid inclusions in pulmonary tuberculosis patient’s sputum samples. The ultrastructure of these cells displayed a triple layered cell envelope which could be clearly distinguished as consisting of an electron dense outer layer (OL), electron transparent layer (ETL), and peptidoglycan layer (PGL) (**Figure 1**). *M. tuberculosis* cells in sputum were identified by the characteristic triple layered cell envelope of mycobacteria and distinguished from other bacteria present in the sputum (**Figures 1A–C**). *M. tuberculosis* cells revealed the accumulation of intracytoplasmic lipid inclusions in sputum sample S1 (**Figure 1A** and **Table 2**). The ETL of the cell envelope had an average thickness of 10.7 nm (±9 nm) in one of the patient sputum sample (S1) and 40 nm (±38 nm) in *M. tuberculosis* cells from another patient sputum sample (S2, **Figure 1B**). This revealed that *M. tuberculosis* cells in human hosts accumulate lipid inclusions and that envelope ultrastructure varies between hosts.

**Table 2:** Quantification of intracytoplasmic lipid inclusions (ILI) in *M. tuberculosis* isolates from the study (*n* ~ 100 cells in each isolate/condition, except S2, *n* = 10 cells).

Growth condition	Sputum (*ex vivo*)		Mid-log (*in vitro*)						
*Mtb* samples	S1	S2	Rv	C1	C2	C3	C4	C5	C6
Average number of ILI per cell	4 (±2)	0	0	2 (±1)	2 (±1)	6 (±3)	4 (±1)	4 (±2)	0
Percentage of cells with ILI	90%	0%	0%	10%	5%	90%	80%	14%	0%
Average size of ILI (nm)	250 (±150)	NA	NA	65 (±25)	100 (±75)	250 (±150)	150 (±50)	120 (±40)	NA

**Stress conditions**	**H_2_O_2_**			**INH**		**DFO**				
***Mtb* samples**	**Rv-H**	**C1-H**	**C4-H**	**Rv-I**	**C1-I**	**Rv-D1**	**Rv-D2**	**C1-D1**	**C1-D2**	**C4-D2**

Average number of ILI per cell	2 (±1)	6 (±3)	4 (±2)	0	4 (±2)	0	5 (±3)	2 (±1)	14 (±7)	11 (±5)
Percentage of cells with ILI	1%	98%	50%	0%	55%	0	99%	15%	100%	100%
Average size of ILI (nm)	70 (±30)	250 (±130)	100 (±40)	NA	80 (±20)	NA	130 (±100)	70 (±50)	140 (±70)	170 (±120)

### Strain-to-Strain Variation in Accumulation of Intracytoplasmic Lipid Inclusions among Clinical *M. tuberculosis* Isolates in Mid-Log Culture Condition

**FIGURE 2:**
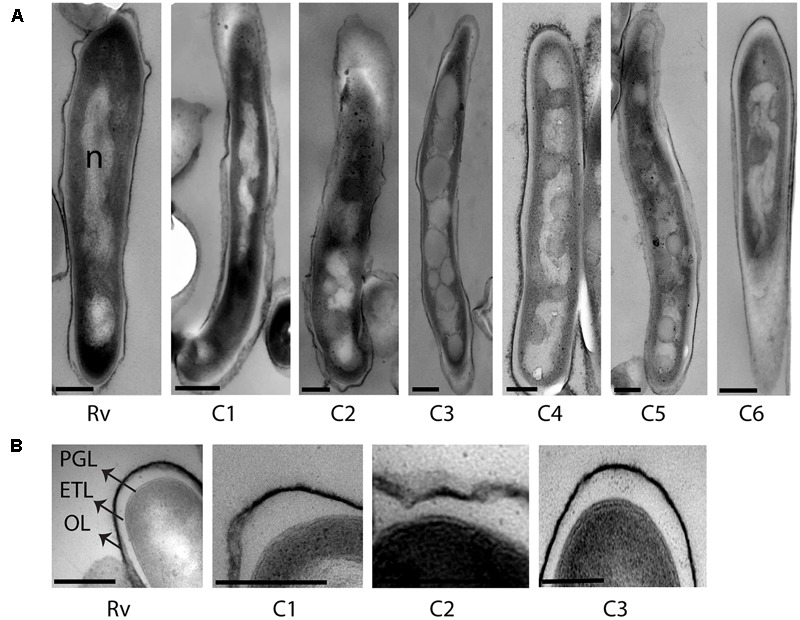
Cellular ultrastructure of clinical *M. tuberculosis* strains under mid-log culture condition. **(A)** TEM images of H37Rv (Rv) and six clinical *M. tuberculosis* strains from mid-log culture (C1–C6). **(B)** TEM images of H37Rv and three clinical *M. tuberculosis* strains (C1–C3) with detailed ultrastructure of cell envelope layers in mid-log culture. OL, outer layer; ETL, electron transparent layer; PGL, peptidoglycan layer; ILI, intracytoplasmic lipid inclusions; n, nucleoid. Scale bar = 200 nm.

We analyzed the cellular ultrastructure of six clinical *M. tuberculosis* isolates (C1–C6) along with H37Rv under mid-log culture condition (**Figure 2A**). Major cellular ultrastructural features of *M. tuberculosis* isolates include the triple layered cell envelope, nucleoid, and cytoplasm. These features were similar in both sensitive (C1, C3, and C5) and resistant (C2, C4, and C6) *M. tuberculosis* isolates (**Figures 2A,B**). We also observed mild (**Figure 2A** and **Table 2**, C1, C2, C4, and C5) to extensive (**Figure 2A** and **Table 2**, C3) accumulation of cytoplasmic lipid inclusions in clinical *M. tuberculosis* isolates, but not in H37Rv and C6 (**Figure 2A** and **Table 2**). All *M. tuberculosis* isolates in mid-log condition had an ETL of average thickness 31.7 nm (±13.1 nm) (Supplementary Figure S1A). We also observed high variation in ETL thickness in the same cell and between different *M. tuberculosis* cells (Supplementary Figure S1A). Based on these *ex vivo* and *in vitro* ultrastructure of clinical *M. tuberculosis* isolates we further analyzed the cellular adaptations under different stress conditions.

### Accumulation of Intracytoplasmic Lipid Inclusions Increased in Oxidative, Iron Deficiency, and Antibiotic Stresses

**FIGURE 3:**
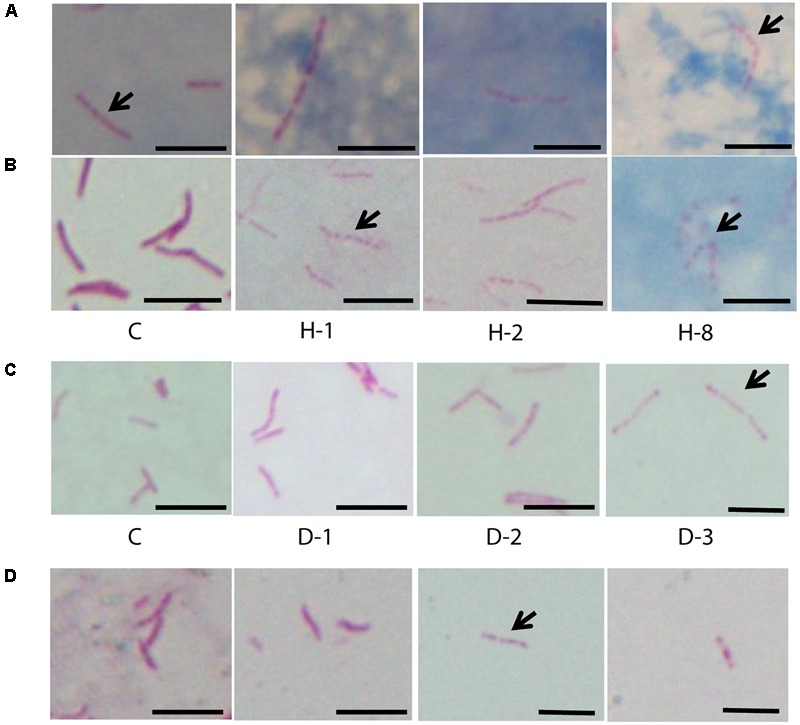
Acid fast staining of *M. tuberculosis* cells in sputum, oxidative stress, iron deficiency, and isoniazid treatment. **(A)** Four sputum samples with *M. tuberculosis* cells (*n* ∼ 100 cells). **(B)** Clinical *M. tuberculosis* strain treated with different concentrations of H_2_O_2_ for oxidative stress and **(C)** DFO for iron deficiency (*n* = 300 cells). **(D)** Clinical *M. tuberculosis* strains grown in the presence of isoniazid (0.015 μg/ml) (*n* ∼ 100 cells). C, untreated control; H_2_O_2_ concentrations used are 21 (H-1), 42 (H-2), and 168 mM (H-8) and the concentrations of DFO are 100 (D-1), 250 (D-2), and 500 μM (D-3), arrow indicates beaded cells and scale bar = 5 μm.

**FIGURE 4:**
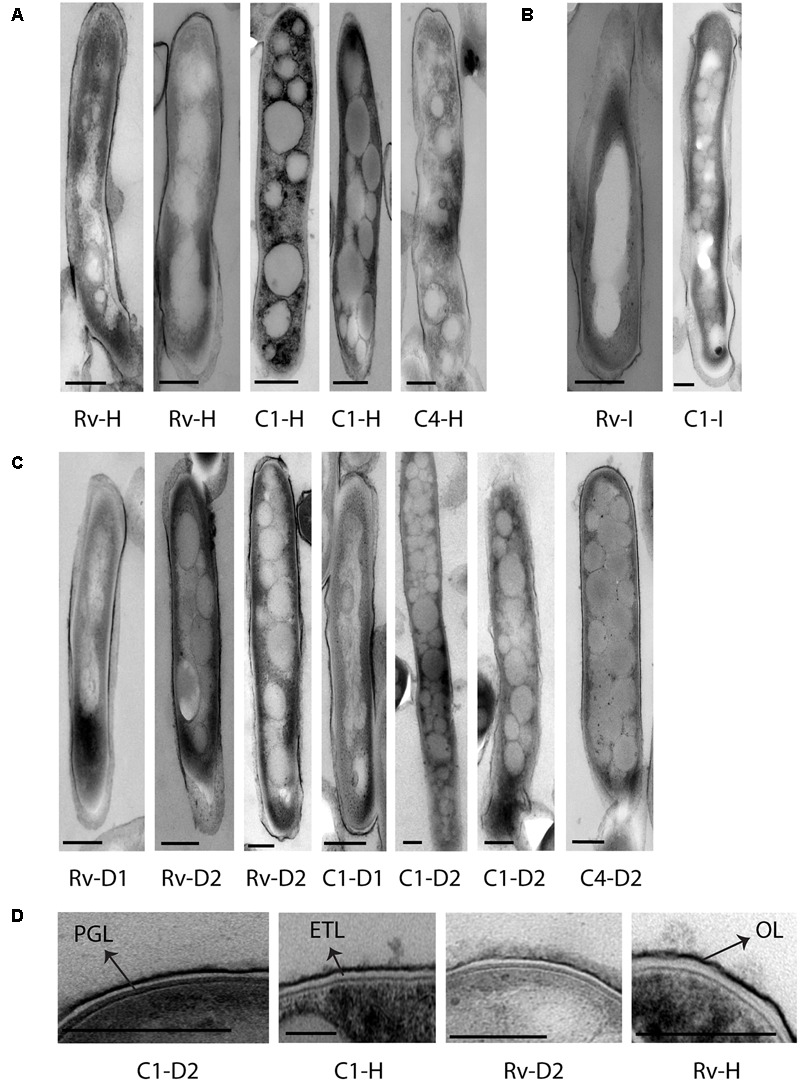
Cellular ultrastructure of *M. tuberculosis* strains under H_2_O_2_, isoniazid treatment, and iron deficiency displaying accumulation of lipid inclusions and thin cell envelope ETL. **(A)** TEM images of H37Rv [Rv-H (two images)] and clinical *M. tuberculosis* strains [C1-H (two images) and C4-H] under H_2_O_2_ treatment. **(B)** TEM image of H37Rv and clinical *M. tuberculosis* strain grown under isoniazid treatment (Rv-I and C1-I). **(C,D)** TEM images of H37Rv and clinical *M. tuberculosis* strains grown under iron deficiency DFO-100 (Rv-D1, C1-D1) and 500 μM [Rv-D2 (two images), C1-D2 (two images) and C4-D2]. DFO, deferoxamine mesylate salt; OL, outer layer; ETL, electron transparent layer; PGL, peptidoglycan layer; scale bar = 200 nm.

We observed *M. tuberculosis* cells with reduced acid fast staining and beaded appearance in sputum, oxidative stress, iron deficiency, and isoniazid treatment (**Figure 3**, *n* ∼ 100–300 cells), and then we characterized the ultrastructure of *M. tuberculosis* under these conditions (**Figure 4**). H_2_O_2_ and isoniazid treatment resulted in a significant accumulation of intracytoplasmic lipid inclusions in clinical *M. tuberculosis* isolate C1 (**Figures 4A,B** and **Table 2**), but not in H37Rv and C4 (**Figures 4A,B** and **Table 2**). H37Rv and clinical *M. tuberculosis* isolates exposed to 100 μM DFO did not accumulate lipid inclusions (**Figure 4C** and **Table 2**) while all isolates treated with 500 μM DFO exhibited accumulation of lipid inclusions (**Figure 4C** and **Table 2**). Both H_2_O_2_ and DFO treatments also resulted in a thinner ETL, with thickness of 13 (±11 nm) and 10.5 nm (± 4 nm), respectively, in *M. tuberculosis* cell envelope as compared to untreated mid-log control (**Figure 4D** compared to **Figure 2B**, *P* < 0.0001 Mann–Whitney *U*-test; Supplementary Figures S1B,C). Similar to the observations in *M. tuberculosis* cells from sputum, different host and antibiotic stresses increased the accumulation of intracytoplasmic lipid inclusions and reduced the cell envelope ETL in *M. tuberculosis* isolates.

### Unique Intracytoplasmic Compartment Observed in *M. tuberculosis* Cells under Iron Deficiency

**FIGURE 5:**
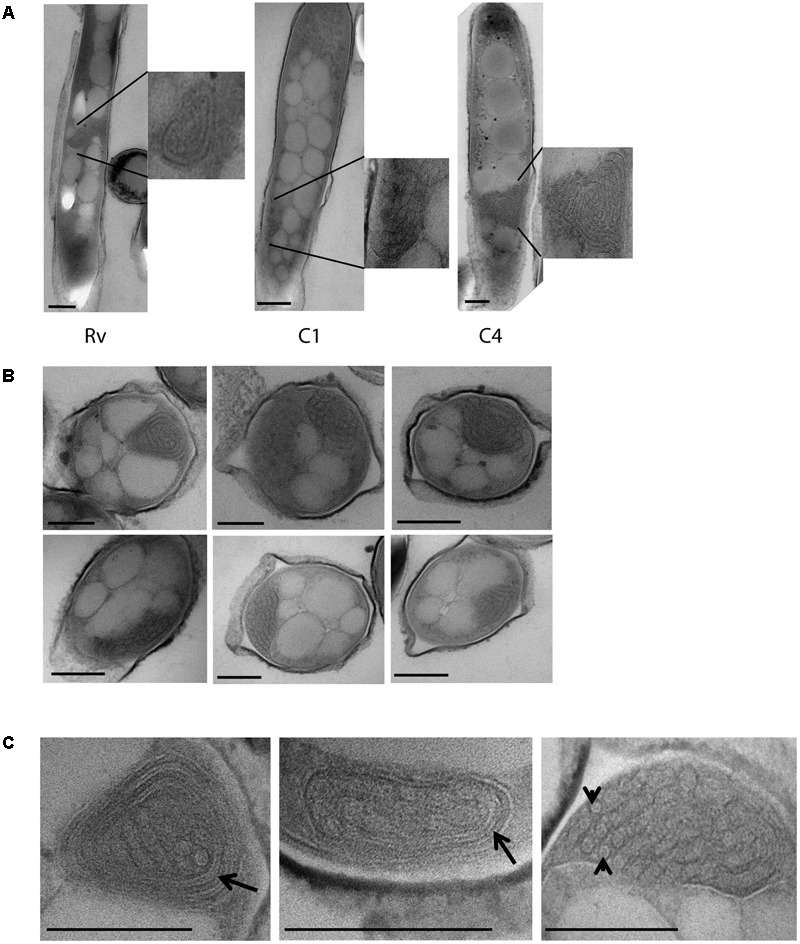
Cytoplasmic compartments in *M. tuberculosis* strains under iron deficiency. **(A)** H37Rv (Rv) and clinical *M. tuberculosis* strains (C1, C4) grown under 500 μm DFO, displaying cytoplasmic compartments in cells along with enlarged images. **(B)** Multiple sections of *M. tuberculosis* cells grown under 500 μm DFO, with cytoplasmic compartment as indicated by asterisks. **(C)** Higher magnification images (40–50 K X) of cytoplasmic compartments from *M. tuberculosis* cells grown under 500 μm DFO, arrow indicates membrane-like structures surrounding the cytoplasmic compartment and arrowhead indicates subunits assembled inside the compartment. DFO, deferoxamine mesylate salt; scale bar = 200 nm.

In addition to the cellular adaptations observed above in different stress conditions, we also observed unique intracytoplasmic compartments in iron-deficient *M. tuberculosis* cells. This compartment was only observed in *M. tuberculosis* grown in the presence of 500 μM DFO and not in cells grown in 100 μM DFO and or the mid-log controls (**Figure 5**). Single intracytoplasmic compartments were observed in all three strains used in this experiment, H37Rv and clinical *M. tuberculosis* isolates (C1, C4), under iron deficiency (*n* = 50 cells observed in each strain) (**Figure 5A**). The average size of this compartment was 250 nm (±50 nm, *n* = 30 cells in total) (**Figure 5B**). At high magnification, we also observed membrane-like structure surrounding these intracytoplasmic compartments, some of which contained small circular units of diameter 17.4 nm (±3.6 nm) (**Figure 5C**).

## Discussion

We analyzed the lipid inclusions and cell envelope layers in clinical *M. tuberculosis* isolates *ex vivo* in sputum representing the host environment. We then compared this with the ultrastructure of clinical *M. tuberculosis* isolates and H37Rv in liquid culture and under different *in vitro* stress conditions. This revealed the accumulation of intracytoplasmic lipid inclusions in clinical *M. tuberculosis* isolates as a cellular adaptation in sputum, liquid culture, and under stress conditions. Analysis of six clinical *M. tuberculosis* isolates revealed isolate-to-isolate variation in the extent of lipid inclusions in mid-log culture and its increased accumulation under stress conditions. The thickness of *M. tuberculosis* cell envelope ETL was significantly reduced under different stress conditions. Formation of an intracytoplasmic compartment in *M. tuberculosis* cells was also observed under iron deficiency.

*Mycobacterium tuberculosis* cells with lipid inclusions have been associated with foamy macrophages and unfavorable treatment outcome in tuberculosis patients (Garton et al., 2002; Peyron et al., 2008; Kayigire et al., 2015; Sloan et al., 2015). In the present study, clinical *M. tuberculosis* isolates displayed lipid inclusions even in liquid culture, which was not observed in the laboratory strain H37Rv. Similarly, *M. avium* and *M. marinum* also do not accumulate lipid inclusions in macrophages and the extracellular environment, respectively (Caire-Brandli et al., 2014; Barisch and Soldati, 2017a). This indicates that accumulation of lipid inclusions is a more prominent cellular adaptation in clinical *M. tuberculosis* isolates compared to laboratory strains of mycobacteria. Supporting this, we also observed increased accumulation of lipid inclusions under both oxidative stress and sub-inhibitory concentration of isoniazid only in clinical *M. tuberculosis* isolates. Isoniazid can also induce oxidative stress and may therefore link these findings (Timmins and Deretic, 2006). It will be interesting to study how other antibiotic treatments influences the accumulation of lipid inclusions in clinical *M. tuberculosis* isolates, as its accumulation may have a role in *M. tuberculosis* persistence to antibiotics (Hammond et al., 2015; Kayigire et al., 2015; Sloan et al., 2015).

We observed increased accumulation of lipid inclusions in *M. tuberculosis* cells at 500 μM DFO compared to 100 μM DFO-treated cells under iron deficiency. DFO concentration-dependent accumulation of lipid inclusions were found in both clinical *M. tuberculosis* isolates and H37Rv. Supporting these observations it has also been reported that iron deficiency and oxidative stress can induce lipid accumulation in mycobacteria, which depends on host foamy macrophages (Bacon et al., 2007; Peyron et al., 2008). Host oxidative stress generates oxidized low-density lipoproteins, and oxygenated mycolic acids present in *M. tuberculosis*; both can trigger the differentiation of host macrophages into foamy cells (Peyron et al., 2008; Palanisamy et al., 2012). This in turn facilitates the accumulation of lipid inclusions in *M. tuberculosis* cells and provides a protective niche for its survival. Our host stress models were based on *in vitro* culture lacking foamy macrophages. Hence, accumulation of lipid inclusions in our host stress models in *M. tuberculosis* cells may have derived lipids from oleic acids present in the culture media, as seen in case of *M. smegmatis* (Garton et al., 2002; Anuchin et al., 2009).

Oxidative stress was also a co-factor in all of the stress conditions where we observed the increased accumulation of lipid inclusions in *M. tuberculosis* cells (Rodriguez and Smith, 2003; Timmins and Deretic, 2006). Transcriptional adaptation of *M. tuberculosis* in macrophages and under *in vitro* stress conditions strongly correlates with the ultrastructural adaptations observed here, indicating that under host stress *M. tuberculosis* shifts to a fatty acid-based metabolism (Schnappinger et al., 2003). Enzymes involved in fatty acid metabolism are also essential for *in vivo* growth and virulence (Munoz-Elias and McKinney, 2005; Reed et al., 2007). The accumulation of lipid inclusions is implicated in *M. tuberculosis* cell division arrest and induction of antibiotic tolerant dormant phenotype (Daniel et al., 2011; Caire-Brandli et al., 2014). This needs to be reinvestigated as our study shows that lipid inclusions *per se* may not inhibit cell division in *M. tuberculosis*. We observed *M. tuberculosis* cells with lipid inclusions growing in mid-log culture and under iron deficiency, similar to the growth observed in *M. marinum* with lipid inclusions (Barisch and Soldati, 2017a). It is possible that accumulation of lipids being a cellular adaptation that can facilitate *M. tuberculosis* entry into, and survival during dormancy (Barisch and Soldati, 2017b).

The unique triple layered cell envelope, reported in several laboratory mycobacterial strains and in clinical strains of *M. tuberculosis* (Takade et al., 1983; Brennan and Nikaido, 1995; Velayati et al., 2009; Vijay et al., 2012), was also observed in all of the clinical *M. tuberculosis* isolates in the present study. The ultrastructure of triple layered cell envelope from our study was also similar to the cell envelope ultrastructure of *M. tuberculosis* processed by cryofixation and rapid freeze substitution (Yamada et al., 2010, 2015). We also observed tearing of resin around *M. tuberculosis* cells in sputum, as observed in TEM images of *M. marinum* granulomas and *M. tuberculosis* cells (Bouley et al., 2001; Vijay et al., 2014). The thickness of the triple layers under mid-log growth conditions was consistent across the six clinical *M. tuberculosis* isolates and H37Rv used here. However, under stress conditions like sputum, oxidative stress, and iron deficiency, we observed a significant reduction in the thickness of cell envelope ETL, although the extent of this reduction varied between the two sputum samples despite a similar bacterial load. These findings suggest that the ETL can be reduced in thickness under host stress, which may vary from patient to patient. This needs to be investigated in a greater number of patients and correlated with aspects such as severity of tuberculosis symptoms and persistence to understand the clinical significance of such adaptations.

The ETL is mainly composed of lipids like mycolic acids (Mdluli et al., 1998; Wang et al., 2000) and transcriptional analysis of *M. tuberculosis* cells under host stress also indicate cell envelope remodeling and fatty acid degradation (Schnappinger et al., 2003). Cell envelope lipids are also involved in host immune modulation and virulence of *M. tuberculosis* strains (Karakousis et al., 2004; Makinoshima and Glickman, 2005). It has also been observed that under different stress conditions *M. tuberculosis* loses acid fastness due to loss of cell envelope lipids and it is associated with dormancy and antibiotic tolerance (Bhatt et al., 2007; Deb et al., 2009). We also observed reduced acid fast staining and *M. tuberculosis* cells with acid fast stained cytoplasmic beads in oxidative stress and iron deficiency. Such cells were also observed in some sputum samples and under isoniazid treatment, these observations strongly correlate with the ultrastructural adaptations such as reduced ETL and accumulation of lipid inclusions in our study. Further investigations are needed to understand the role of reduced cell envelope lipids on the accumulation of intracytoplasmic lipid inclusions in *M. tuberculosis*.

Reduction in the envelope lipids may enhance the permeability of cell envelope and influence the susceptibility of *M. tuberculosis* to antibiotics. Cell envelope modifications and enhanced antibiotic susceptibility in *M. smegmatis* have been observed under iron deficiency (Pal et al., 2015). Triacylglycerol is also a component of *M. tuberculosis* cell envelope and loss of acid fastness is observed under iron deficiency and in hypoxia (Rastogi et al., 2017). Such cell envelope modifications accompany non-replicative persistence and antibiotic tolerance of *M. tuberculosis in vitro* (Rastogi et al., 2017). These observations indicate the influence of host factors on cellular adaptations in *M. tuberculosis* and antibiotic susceptibility. As there are multiple host factors and complex interactions influencing antibiotic susceptibility, this needs to be investigated further to identify the factors that can enhance susceptibility to antibiotics. There was significant variation in the accumulation of lipid inclusions in clinical *M. tuberculosis* isolates in mid-log culture. Such differences between *M. tuberculosis* isolates may have a clinical significance in persistence against host stress and antibiotics. Hence, variations in cellular adaptations need to be correlated with persistence and antibiotic tolerance among clinical *M. tuberculosis* isolates to understand its role in treatment failure.

Beijing lineage was shown to accumulate triacylglycerides and has triacylglyceride synthase gene (Rv 3130c) upregulated during *in vitro* growth (Reed et al., 2007). This gene is a member of DosR regulon, and some of the regulon genes are constitutively overexpressed in Beijing lineage (Domenech et al., 2017). DosR and WhiB3 have been shown to modulate lipid accumulation in *M. tuberculosis* (Singh et al., 2009), and also contribute to bacilli adaptation to hypoxia and redox stresses, respectively (Park et al., 2003; Saini et al., 2004; Singh et al., 2009). These proteins may play a role in the accumulation of lipid inclusions under oxidative stress and iron deficiency in clinical *M. tuberculosis* isolates. The mechanism of formation of lipid inclusions in mycobacteria also involves interactions with host lipid droplets and membrane phospholipids (Barisch and Soldati, 2017b). Thus, host stresses may induce significant cell biological adaptations in clinical *M. tuberculosis* isolates; its molecular mechanism needs to be further investigated.

In addition to reduction in the thickness of ETL and accumulation of lipid inclusions in *M. tuberculosis* cells, we also observed intracytoplasmic compartments under iron deficiency. These compartments were approximately 200 nm in size and were specifically observed in all *M. tuberculosis* isolates cultured under 500 μM DFO. It is possible that these compartments are mesosomes as observed in bacteria treated with antibiotics (Santhana Raj et al., 2007; Li et al., 2008). Studies have also shown the formation of intracellular compartments which accumulate H_2_O_2_ under cellular damage (Ebersold et al., 1981; Li et al., 2008; Xin et al., 2014). Mesosomes and other such intracellular structures are considered as ultrastructural artifact induced under chemical fixation and dehydration process, and these are not observed under cryo-electron microscopy lacking such fixation (Pilhofer et al., 2010). In this study we have used primary fixation with osmium tetroxide for 1 h, and post-fixation with glutaraldehyde for 2 h. There is a possibility of such chemical fixation inducing the formation of intracellular structures, specifically under stress conditions. Cellular adaptations under iron deficiency may increase the probability of formation of such structures during chemical fixation, as we observed them only in *M. tuberculosis* cells under iron deficiency. In cryoelectron microscopy cells are imaged at frozen-hydrated state without chemical fixation or dehydration of cells and can avoid much of the fixation artifacts (Pilhofer et al., 2010). Cytoplasmic structure termed as stack has been reported in slow growing *Pseudomonas deceptionensis* M1 by TEM and also confirmed by cryo-electron microscopy (Delgado et al., 2013). If confirmed to be a true cellular structure by cryoelectron microscopy and specific for *M. tuberculosis* in iron deficiency. These compartments probably may have a role in iron storage.

Iron limitation has been a common host defense encountered by *M. tuberculosis*; hence, it has evolved mechanisms to sequester iron from the host by using siderophores like mycobactin (Rodriguez and Smith, 2003; Ratledge, 2004). Inside *M. tuberculosis* cells bacterioferritins BfrA and BfrB function as iron storage proteins. Recent observations have shown that BfrB can be encapsulated by the protein encapsulin to form nanocompartments *in vitro* (Reddy et al., 2012; Contreras et al., 2014). We observed arrangement of units with size ∼20 nm inside these intracytoplasmic compartments in *M. tuberculosis* cells, which is similar in size to the encapsulin observed *in vitro* (Contreras et al., 2014). These observations suggest that these intracytoplasmic compartments may be encapsulin-based nanocompartments in *M. tuberculosis*. They may be used to isolate excess of iron from generating oxidative cellular damage or a similar protective function under stress (Contreras et al., 2014). Iron storage has been essential for *M. tuberculosis* survival and virulence, hence has also been a potential novel drug target (Pandey and Rodriguez, 2012). It is important to investigate further the nature of these intracytoplasmic compartments in avirulent laboratory strains by cryo-electron microscopy and its role in *M. tuberculosis* survival under iron deficiency. Recent observations further implicate survival of *M. tuberculosis* in iron deficiency and accumulation of lipid inclusions to antibiotic tolerance and persistence (Baron et al., 2017; Kurthkoti et al., 2017).

In summary, we were able to demonstrate the major cellular adaptations of clinical *M. tuberculosis* isolates to host and antibiotic stress conditions. Further investigation of these cellular adaptations and their role in *M. tuberculosis* survival under stress is important. These will aide in our understanding of the ability of *M. tuberculosis* cells to persist during host and antibiotic stress. The variations in cellular response among clinical *M. tuberculosis* isolates may be associated with the persistence and treatment outcome among patients.

## Author Contributions

SV, NT, GT, NP, and EJ conceived and designed the experiments. SV, HH, and DT did the experiments. SV and AP did TEM analysis. SV, NT, GT, EJ, and AP analyzed and interpreted the data. SV, HH, DT, NP, NT, GT, AP, and EJ drafted and revised the manuscript and approved the final version.

## Conflict of Interest Statement

The authors declare that the research was conducted in the absence of any commercial or financial relationships that could be construed as a potential conflict of interest.
