# Supplementary figures and images for: Ultrastructural Analysis of Cell Envelope and Accumulation of Lipid Inclusions in Clinical Mycobacterium tuberculosis Isolates from Sputum, Oxidative Stress, and Iron Deficiency
Source: Front Microbiol. 2018 Jan 11;8:2681. doi: 10.3389/fmicb.2017.02681 (PMC5770828; doi:10.3389/fmicb.2017.02681)

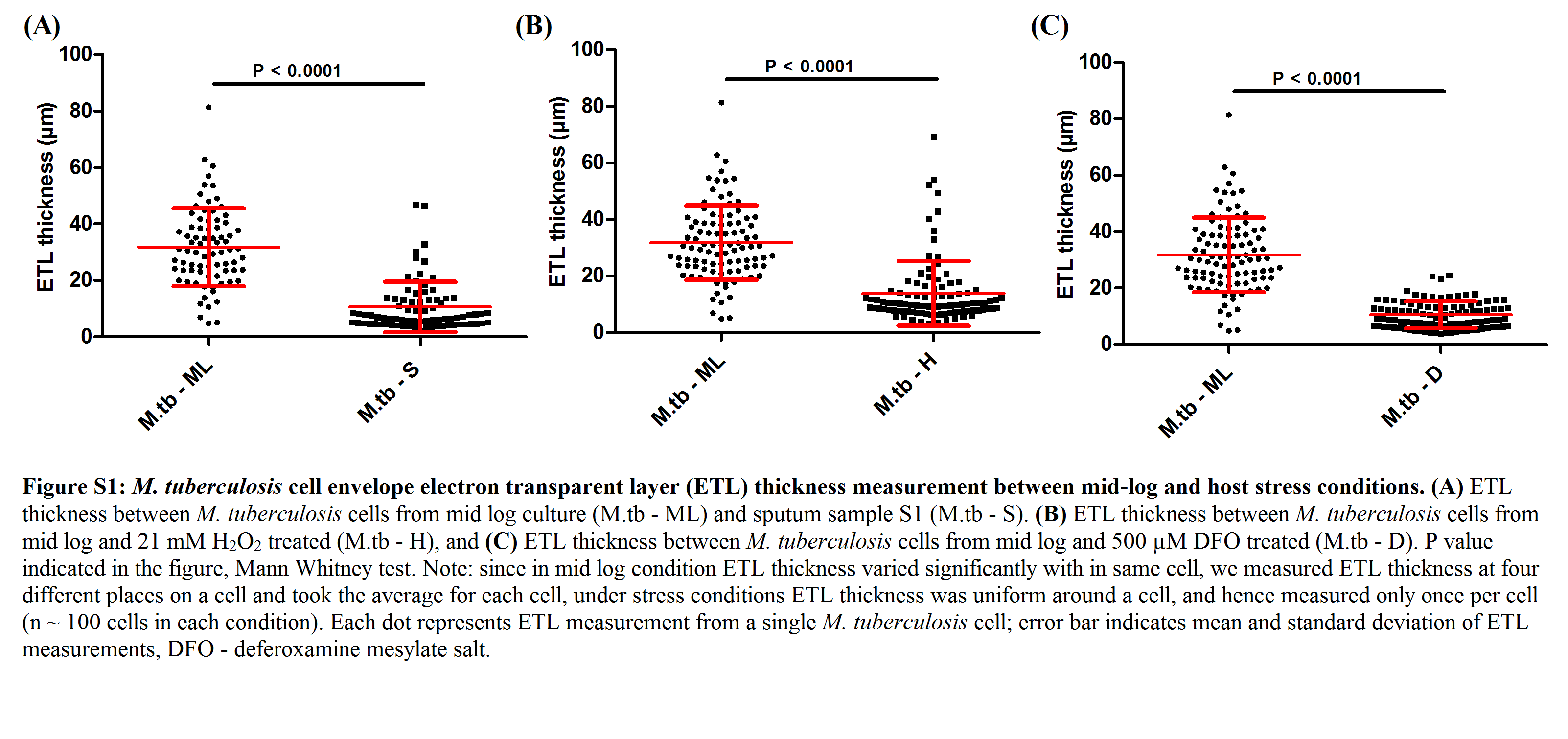

Supplement: Supplementary file 1 [file Image_1.TIF]
